# Spatiotemporal dynamics of mammalian wound healing

**DOI:** 10.1038/s41421-025-00865-2

**Published:** 2026-01-14

**Authors:** Julià Agramunt, Yuanbo Kang, Yuval Rinkevich

**Affiliations:** 1 Institute for Regenerative Biology and Medicine, Chinese Institutes for Medical Research, Beijing, China; 2 Department of Plastic Surgery, Peking Union Medical College Hospital, Chinese Academy of Medical Sciences, Beijing, China; 3 Capital Medical University, Beijing, China

**Keywords:** Mechanisms of disease, Cell migration

## Abstract

Mammalian wound healing is orchestrated by tightly regulated cellular and molecular programs across the hemostasis, inflammation, proliferation, and remodeling phases. Here, we propose the concept of spatiotemporal clocks as a unifying framework for understanding how transitions between phases are coordinated. We dissect the roles of distinct spatial domains: epidermis, dermis, fascia, wound edges, and wound center, and highlight the oscillatory molecular signals that govern their dynamic interactions. Special attention is given to wound-induced hair neogenesis (WIHN) as a model of regenerative potential. By integrating spatial and temporal dimensions, this framework unifies the multidimensional aspects of wound healing, laying a robust foundation for the development of innovative therapeutic strategies.

## Introduction

Throughout their lifetimes, organisms are exposed to a vast array of damaging stimuli, ranging from microbial pathogens such as viruses and bacteria to physical threats posed by conspecifics, predators, and environmental hazards. These challenges often result in injuries that, if not properly addressed, can lead to death. In mammals, the skin serves as the first line of defense, protecting the body from infections, external irritants, and traumatic injuries. When this barrier is breached, the body relies on complex mechanisms to heal wounds and regenerate damaged tissues. When injured, epithelial barriers such as skin trigger an evolutionarily conserved wound healing process, as observed in *C. elegans*^1,2^, with distinct yet overlapping temporal phases: hemostasis, inflammation, proliferation, and remodeling^3^. Each phase is characterized by specific molecular and cellular dynamics that define its function and outcomes.

Hemostasis, initiated immediately after injury, triggers vasoconstriction and activates the clotting system, driven by platelets that start the coagulation cascade and deposit fibrin to prevent blood loss and shield against microbial infection^4–9^. Within the wound bed, the deposition of extracellular matrix (ECM) components, coupled with an increased gradient of platelet-derived growth factors and other molecules, triggers angiogenesis. Here, new capillaries proliferate rapidly, forming an extensive network of blood vessels that is denser than that of normal tissue^10^. The initiation of angiogenesis is positively regulated by several soluble factors, with vascular endothelial growth factor A (VEGF-A) being the most prominent. In addition to VEGF-A, other factors such as fibroblast growth factor-2, platelet-derived growth factor, members of the transforming growth factor-beta (TGF-β) family, and cardiac ankyrin^11–13^. These events promote cell migration and the recruitment of the immune system, setting the stage for subsequent healing phases^14^.

**Fig. 1:**
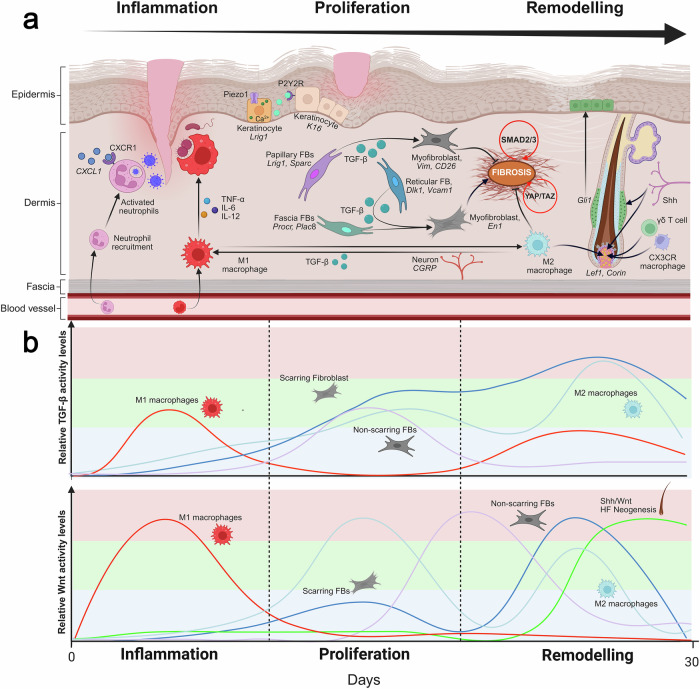
Spatiotemporal dynamics of wound healing across inflammatory, proliferative, and remodeling phases. **a** Temporal overview of cutaneous wound healing through inflammatory, proliferative, and remodeling phases (black arrow, top). During the inflammatory phase, platelets recruit immune cells (not shown), and CXCL1 activates neutrophils via CXCR1 receptors to clear pathogens, including viruses. M1 macrophages are activated through TNF-α, IL-6, and IL-12 signaling. In the early proliferative phase, distal stem cell keratinocytes expressing Lrig1 are activated by tension signals from Piezo1 channels, increasing Ca²⁺ gradients and releasing ATP, which stimulates wound-edge keratinocytes via P2Y2 receptors to migrate and re-epithelialize the wound in the epidermis. In the dermis, fibroblast populations—papillary (Lrig1, Sparc), reticular (Dlk1, Vcam), and fascia (Procr, Plac8) — are activated by TGF-β signaling and differentiate into myofibroblasts. Papillary fibroblasts adopt a non-scarring phenotype (Vim, CD26), while fascia and reticular fibroblasts differentiate into scarring En1 fibroblasts. Late in the proliferative phase, M1 macrophages transition to a non-inflammatory M2 phenotype via TGF-β and neuronal CGRP-mediated signaling to minimize scarring. In the remodeling phase, Smad and YAP/TAZ pathways regulate fibroblast activity. In large wounds, wound-induced hair neogenesis (WIHN) occurs late in remodeling, driven by Shh/Wnt signaling, γδ T cells, and CX3CR1 macrophages, alongside M2 macrophages, promoting dermal condensate formation and new DP expressing Lef1 and Corin, triggering de novo HF neogenesis. Shh signaling also activates Gli1 HF stem cells, which can acquire an epidermal phenotype and contribute to wound re-epithelialization and remodeling. **b** Spatiotemporal dynamics of TGF-β (top) and Wnt (bottom) signaling levels across cellular niches during wound healing phases, from the inflammatory phase (day 0) to the end of the remodeling phase (day 30). Lines represent independent relative levels for each cell type in 3 different activation zones: red high, green moderate, blue low.

The inflammatory phase begins within 24 hours of injury and may last for weeks in normal wounds, or longer in chronic non-healing wounds^15^. Mast cells release enzymes, histamine, and amines while pro-inflammatory M1 macrophages clear infection and debris, releasing pro-inflammatory cytokines and growth factors^16–18^. The transition from the inflammatory to the proliferative phase is tightly regulated and involves a complex interplay of immune and non-immune cells, as well as molecular signals that resolve inflammation and initiate tissue repair^19^. This transition is primarily driven by a shift in macrophage phenotypes from pro-inflammatory M1 to reparative M2, a process triggered by anti-inflammatory cytokines such as IL-4 and IL-10, glucocorticoids, and prostaglandins^20–22^. Stromal cells also contribute by regulating immune cell apoptosis, aiding in the resolution of inflammation^23^. During the proliferative phase, fibroblasts transition into myofibroblasts, depositing collagen-rich ECM and transferring into wounds a composite fascia connective tissue that forms a matrix primordium within the wound bed with marked angiogenesis^24–27^. Re-epithelialization, a hallmark of the proliferative phase in mammalian wound healing, restores the epidermal barrier through intricate keratinocyte dynamics via TGF-β1 and Wnt/β-catenin signaling^28^. Keratinocytes at the wound periphery undergo phenotypic shifts, upregulating integrins to engage the ECM and facilitate migration^29,30^. Finally, the remodeling phase restructures the newly formed tissue through the reorganization of the ECM, reduction in cell density, and maturation of scar tissue^31^. The remodeling phase critically shapes healing outcomes, driven by dynamic interactions between immune cells and diverse fibroblast populations across distinct wound regions^19,24,31–38^. A key feature during the remodeling phase in murine wound healing is wound-induced hair neogenesis (WIHN), where the hair follicle (HF) regenerates in healed wound areas, particularly in large wounds^39,40^. WIHN is driven by molecular and mechanical cues such as Wnt/β-catenin signaling pathway and ECM tensions^41–46^. At the end of the remodeling phase, the outcome of the healing process varies depending on the injury’s severity and the dynamics of the healing response which determines whether the result is scarless regeneration or fibrotic repair^33,47,48^ (Fig. 1a). The former may result in healthy, functional skin, while the latter can lead to non-functional skin, potentially causing hypertrophic scars and keloid scars in humans^49^. If the skin fails to heal properly, the restoration of tissue integrity can be compromised, leading to the development of chronic wounds such as ulcers^50–52^.

In addition to the temporal phases, a spatially organized architecture of cells and regions is intrinsically important for the wound healing process^53–57^. Common anatomical regions such as the epidermis, papillary dermis, reticular dermis, and fascia have been shown to exhibit distinct vascularization, transcription, and molecular signaling processes^58,59^. The edges of the wound and its center also display distinct cellular profiles and molecular events^57,60^. Furthermore, differences in wound size have also been shown to display distinct molecular and cellular profiles^57,61,62^. Thus, both the temporal phases and wound spatial architecture orchestrate the molecular and cellular events that dictate healing progression (Fig. 1b).

Building on the growing body of recent research, this review aims to illustrate how mammalian wound healing operates as a complex spatiotemporal program, orchestrated across distinct phases and wound architectures to restore tissue integrity. We highlight the temporal progression, particularly the proliferative and remodeling phases and examine its spatial domains, including dermis, epidermis, fascia, wound edges, wound center, and wound size, each harboring specialized cellular niches. We then analyze how cell fate, immune responses, and mechanical tension dynamics converge through spatiotemporal cues. Finally, we propose the concept of spatiotemporal clocks as a unifying framework to understand wound healing dynamics.

## Spatiotemporal clocks as regulators of wound healing dynamics

Several theories have been developed to explain how temporal and spatial information are integrated in biological processes. The French flag model, introduced by Lewis Wolpert in the 1960s, describes how morphogens create concentration gradients that dictate cell fates based on their position within a tissue^63^. In this model, cells interpret molecular gradients to activate specific genes at distinct concentration thresholds. This framework has been validated across fields like biochemistry, embryogenesis, and spatial transcriptomics^64–67^. More recent models have built upon it by incorporating robust temporal dynamics. For example, the clock-dependent oscillatory gradient model introduces oscillators molecular or cellular mechanisms that produce fluctuations in gene expression and protein signaling to integrate time and space^68,69^. In skin biology and wound healing, spatiotemporal oscillators are emerging as critical regulators of skin repair and wound healing. For example, ERK waves function as such oscillators by propagating across epithelial sheets to coordinate the direction and timing of collective cell migration. These oscillatory signals synchronize actomyosin contraction and modulate cell density, thereby aligning local biochemical inputs with large-scale mechanical outputs necessary for wound closure^70^.

The complex spatiotemporal interplay of cellular and molecular elements, combined with lineage heterogeneity and diverse functional roles, presents significant challenges to fully understanding tissue repair dynamics. To address this, we introduce spatiotemporal clocks: biological timing mechanisms that orchestrate mammalian wound healing by synchronizing temporal and spatial dynamics. This framework integrates molecular oscillators, such as key components of the TGF-β and Wnt signaling pathways, which regulate such events across distinct wound healing phases. These oscillatory mechanisms modulate immune responses, cell migration, and tissue remodeling by facilitating phase transitions. For example, TGF-β signaling exhibits peak activity during the inflammatory phase within the M1 macrophage niche. When activated in conjunction with IL-10, it triggers the transition from M1 to M2 macrophages, which subsequently recruits fibroblasts to initiate the proliferative phase^32,71–73^. Divergent functional outcomes may also arise depending on signaling levels. Elevated TGF-β levels in both M1 and M2 macrophages could disrupt healing, potentially sustaining inflammation through M1 macrophage dominance or inducing M2-to-M1 transitions, which may lead to scarring or chronic wounds^18,32,72^. Thus, the activation of molecular oscillators within specific wound healing phases and regions establishes a spatiotemporal clock that regulates phase transitions. This clock may facilitate or impede normal progression between wound healing phases, thereby influencing outcomes. Precise coordination can promote favorable outcomes, such as tissue regeneration, while dysregulation may lead to adverse outcomes, including fibrosis or ulceration. In the following sections, we will analyze the spatiotemporal dynamics of wound healing to further develop this framework and provide specific examples of its application to current research findings.

## Wound healing dynamics: temporal complexity

Each of the wound healing phases is governed by molecular programs that evolve dynamically over time^15^. Major molecular pathways are involved in phase-specific roles^74–78^. While most pathways influence multiple phases, their key effector molecules reveal phase-specific dominance and cross-regulation^79,80^.

TGF-β has been identified as one of the first pathways recruited to the injury site by platelets^4,5^. Ex vivo studies with mice have shown that TGF-β levels peak immediately after injury, decrease in the following days, and rise again by day 5 post-injury, suggesting the oscillatory nature of TGF-β and its importance for the initiation of the inflammatory phase and the proliferative phase^81^. During proliferation, TGF-β — secreted mainly by anti-inflammatory M2 macrophages that stimulates fibroblast migration, proliferation, and differentiation into myofibroblasts^25^. These myofibroblasts then produce ECM components (e.g., collagen, fibronectin), forming granulation tissue, promoting wound contraction, and supporting angiogenesis via macrophage-derived VEGF^32^. In the remodeling phase, TGF-β enhances ECM deposition and inhibits matrix metalloproteinases (MMPs), ensuring tissue maturation and collagen realignment^82^. Different fibroblast subtypes respond variably to TGF-β isoforms (e.g., TGF-β1 upregulates ACTA2 (α-SMA), while TGF-β3 may attenuate signaling), influencing the healing process^83^.

There is significant crosstalk among molecular signals and pathways during wound healing, adding layers of coordination. For example, in the hemostasis and inflammation phases, Wnt/β-catenin signaling facilitates initial repair responses. For example, Wnt3A, promotes megakaryocyte proliferation and proplatelet formation, enhancing platelet production^84,85^. Platelets release growth factors such as PDGF which recruit monocytes that differentiate into pro-inflammatory M1 macrophages^86,87^. These macrophages secrete cytokines and Wnt5a which acts as a positive feedback loop driving pro-inflammatory M1 to anti-inflammatory M2 macrophage transition^88–90^. This switch, mediated by target cytokines and genes like IL-10 and ARG1 respectively, curbs excessive inflammation and sets the stage for the subsequent healing phase^91^. In the proliferative phase, Wnt/β-catenin signaling is critical for re-epithelialization, angiogenesis, and connective tissue formation. By activating genes like CCND1 (in epithelial cells) and VEGFA (in endothelial cells), it promotes stem cell differentiation and tissue repair^92^. Wnt16 activation has been shown to promote keratinocyte proliferation for wound closure and angiogenesis^93,94^. Fibroblasts, stimulated by Wnt3A, produce ECM components (e.g., fibronectin via fibronectin 1), forming connective tissue^95^. In the remodeling phase, Wnt3a and Wnt5a induce fibroblast-to-myofibroblast differentiation mediated by cytoskeletal disorganization, align collagen fibers and promote fibrosis^96–98^. Furthermore, Wnt/β-catenin signaling also promotes fibrosis by blocking adipogenic differentiation of dermal fibroblasts, reducing adipocyte formation, and enhancing ECM deposition^99^.

Finally, the Wnt/β-catenin pathway has been implicated in WIHN during the late remodeling phase. Intriguingly, the induction of Hair Follicles (HFs) in this phase is mediated by crosstalk Shh/Wnt signaling^100,101^. Epidermal overexpression of Shh drives extensive HF neogenesis by activating the formation of a neogenic dermal condensate, which gives rise to the dermal papilla (DP)^102,103^. This subsequently induces the K15+ epithelial outer root sheath and ultimately forms a mature HF. While prolonged Wnt/β-catenin activation is associated with fibrotic outcomes (e.g., excessive ECM deposition and myofibroblast differentiation), Shh signaling in Wnt-active cells redirects dermal fibroblasts toward a DP fate^39^. This suggests a synergistic interplay between Wnt and Shh pathways in the late remodeling phase: Wnt primes fibroblasts for plasticity, while Shh specifies regenerative DP differentiation, thereby converting wounds into regenerative microenvironments.

Thus, the temporal regulation of key pathways like TGF-β and Wnt/β-catenin suggests that wound healing is not progression of isolated phases, but rather a dynamic interplay of molecular events regulated by tight oscillatory patterns. These pathways exhibit phase-specific dominance: different TGF-β isoforms drive early inflammation and later fibrosis, while Wnt/β-catenin coordinates proliferation and remodeling, yet their roles are non-linear, overlapping, and interdependent. However, a critical question arises: how do these temporally entangled signals resolve into coherent tissue repair? The answer may lie in the spatial regulation of pathway activity where ligands, receptors, and effectors are localized to specific wound areas (e.g., wound edge vs center or fascia vs dermis) and cellular niches.

## Wound healing dynamics: spatial complexity

Wound healing is shaped by the spatial arrangement of skin layers (epidermis, dermis, fascia) and wound regions (edge vs center), as well as specialized cellular microenvironments or niches where specific cell types interact with molecular and mechanical cues^104–108^.

### Epidermis and keratinocytes in wound healing

In the epidermis, keratinocytes follow a spatially organized dynamics that dictates their proliferation, migration, and intercellular signaling^30,41,109^. Adjacent to the wound margin, a proliferative zone emerges within hours of injury, initiated by mechanosensitive signaling which activates ERK1/2 phosphorylation and cyclin B transcription to promote mitosis and a peak of Wnt/β-catenin activity^54,110–112^. Spatial coordination is further mediated by paracrine Ca²⁺ signaling, which orchestrates collective keratinocyte behavior: mechanical stress at the wound edge activates, PIEZO1 channels, triggering intracellular Ca²⁺ transients that propagate to adjacent cells via ATP release through connexin or pannexin hemichannels^108^. Extracellular ATP engages purinergic receptors, such as P2Y2, on neighboring keratinocytes, eliciting secondary Ca²⁺ influx and establishing a wave-like signal that synchronizes migration and proliferation across zones. This Ca²⁺ gradient maintains spatial segregation: leading-edge migratory K16+ keratinocytes exhibit sustained Ca²⁺ oscillations that drive actomyosin-mediated migration, whereas distal proliferative stem cell keratinocytes expressing Lrig1 experience transient Ca²⁺ pulses that enhance cyclin-dependent kinase activity^113^. Disruption of this signaling, through inhibition of purinergic receptors or connexin channels, compromises collective migration and zone coordination^114–121^. At the migratory-edge front, keratinocytes undergo significant molecular remodeling. Leading-edge cells downregulate hemidesmosomal α6β4 integrins, essential for basement membrane adhesion, while upregulating β1 integrins (α5β1, αvβ6) to engage the fibrin-rich provisional matrix of the wound bed^122–126^. This partial epithelial-mesenchymal transition enables cell rearrangement and tension redistribution, advancing the epidermal front. Matrix metalloproteinase-1 (MMP1) secretion further supports migration by degrading collagen and fibrin barriers within the wound matrix^127–129^. Furthermore, keratinocytes are also involved in modulating the immune response by recruiting immune cells such as neutrophils and macrophages. For example, in response to wounding, keratinocytes release the chemokine MCP-1, which attracts macrophages and T cells to the injury site^130,131^. In summary, keratinocyte wound re-epithelialization is governed by spatially and temporally segregated mechanisms: in the beginning regulators of the proliferative phase, Wnt/β-catenin and ERK triggers paracrine mechanoactivated Ca²⁺ signaling that synchronizes proliferation and migration in the wound edge while proliferative cells show pulsatile Ca²⁺ dynamics to control progenitor cell migration.

### Dermis, fascia and cell migration: spatial dynamics form specific cellular niches

Below the epidermis, the dermis and the fascia are spatially complex layers, each hosting distinct fibroblast populations^47,48,132–134^. The dermis comprises the papillary dermis (upper, loose connective tissue) and the reticular dermis (deeper, dense collagen), while the fascia, a much deeper connective tissue layer, supports rapid matrix deployment^34,135,136^. These layers exhibit spatial and cellular heterogeneity, influencing fibroblast behavior across wound zones and throughout the healing process.

Single-cell RNA-seq analyses have elucidated distinct fibroblast subpopulations, each characterized by classical and subtype-specific molecular markers that serve as spatial-localization cues dictating their functional specialization and fate (reviewed in Torregrossa M et al., 2025). Platelet-derived growth factor receptor alpha (PDGFRα), a pan-fibroblast marker, broadly identifies fibroblasts, while additional markers delineate subtypes with specialized roles: papillary fibroblasts, expressing Sparc, Col3a1 and Lrig1, contribute to structural maintenance and tissue remodeling; reticular fibroblasts, marked by Dlk1, Cxcl12, and Vcam1, support migratory and contractile functions essential for early wound healing; fascia fibroblasts, defined by Plac8, Procr, Sfrp2, and TGF-β2, mobilize ECM to expedite repair; and myofibroblasts, a differentiated state arising from these subtypes, drive ECM deposition and wound contraction through markers such as Postn, Acta2 (ɑ-SMA) and En1 often contributing to fibrosis^83,137^. The spatial organization of fibroblasts across dermal and fascial layers is not merely structural but reflects functional niches where location-specific gene expression governs lineage commitment. For instance, fascia fibroblasts reside in ECM-rich niches primed for rapid repair, whereas papillary fibroblasts in the dermis express genes tailored for matrix remodeling^83^. This intricate interplay between spatial context and marker expression suggests that fibroblast identity and fate are intrinsic and lineage-restricted. Reciprocal transplantations of scar-producing fibroblasts (Engreiled-1 lineage positive fibroblasts) from backskin into buccal cavity, and of scar-neutral fibroblasts (Wnt1-lineage positive fibroblasts) from buccal cavity into backskin, culminated in ectopic dermal architectures that mimic their place of origin, indicating these are lineage-specific and cell-intrinsic functions that have been set during embryonic development^34^.

Following injury, fibroblasts from the dermis and fascia migrate to the wound bed. This migration is spatially coordinated: at the wound edge, fibroblasts adopt a migratory phenotype driven by chemotactic gradients of PDGF and TGF-β signaling^138^. These signals activate integrins such as α5β1, enabling adhesion to the provisional fibrin matrix and directing fibroblasts toward the wound center^124,139^. In the wound center, fibroblasts shift to a proliferative and matrix-synthetic role, producing collagen and other ECM components to rebuild tissue^47^. Mechanical tension further shapes this spatial segregation: higher tension at the wound edge promotes myofibroblast differentiation via RhoA/ROCK signaling, while lower tension in the center supports ECM synthesis^47,140^. Fascia fibroblasts exhibit a unique behavior termed fascia swarming, where they collectively migrate as cohesive sheets, facilitated by N-cadherin-mediated cell–cell adhesion^141^. This process delivers prefabricated ECM to the wound, accelerating repair and highlighting the fascia’s role in wound healing. Thus, wound healing in the dermis and fascia is governed by spatially segregated mechanisms: chemotactic gradients, fibroblast types and mechanical cues drive fibroblast migration and differentiation. Papillary and reticular fibroblasts in the dermis, alongside fascia fibroblasts, coordinate their activities across wound zones to restore tissue integrity.

### Cells with multispatial presence

Despite the critical roles of fibroblasts and keratinocytes in the spatiotemporal regulation of wound healing, there are also other relevant cell types that are distributed across the entire wound area. For example, neurons regulate tissue repair through intricate neuro-immune interactions. Nociceptive sensory neurons, specifically those expressing Naᵥ1.8, promote skin wound repair and muscle regeneration post-injury via the neuropeptide calcitonin gene-related peptide (CGRP). CGRP signals through receptor activity-modifying protein 1 (RAMP1) on immune cells like neutrophils and macrophages, modulating their recruitment, survival, and polarization towards a pro-repair phenotype, partly mediated by thrombospondin-1^142^. In mice lacking nociceptors or in diabetic models with peripheral neuropathy, wound healing is impaired, but delivering engineered CGRP enhances repair, highlighting the therapeutic potential of leveraging neuronal signaling^142,143^.

Schwann cells, originating from disrupted dermal nerves, migrate into granulation tissue, dedifferentiate, and proliferate post-injury. Genetic ablation of Schwann cells delays wound closure, reduces myofibroblast formation, and impairs re-epithelialization, while their expansion via Pten inactivation enhances TGF-β signaling and myofibroblast differentiation^144,145^.

Pericytes, traditionally viewed as microvascular stabilizers, play dynamic roles in cutaneous wound healing across its phases, from coagulation to proliferation. Beyond regulating vessel tone and angiogenesis, they detach during early repair to secrete TGF-β and MMPs, facilitating endothelial migration, and later clear excess vasculature via CXCR3-mediated signaling^146,147^. Recent findings reveal their role in mediating vascular responses to fibrotic ECM changes. In fibrotic collagen environments, pericytes exhibit abnormal migration, reduced differentiation, and increased inflammatory gene expression, driven by NOTCH3-dependent signaling^148^. Silencing NOTCH3 abolishes this response, its role in endothelial-pericyte crosstalk^146^.

### Wound architecture dictates regeneration or repair

Wound size is another intrinsic factor influencing the healing process: larger wounds exhibit greater regenerative potential, similar to embryonic tissues whereas wounds heal by scarless regeneration^149^. The dynamics of this process are poorly understood, but recent advances have revealed that several factors contribute to this enhanced regenerative potential. Firstly, the prolonged healing time of large wounds due to their size extends the window for regenerative signaling, allowing sustained activation of pathways critical for tissue renewal^150,151^. This extended duration may enable the persistence of inflammation and immune responses that favor regeneration over fibrosis. For instance, immune cells like γδ T cells, which secrete fibroblast growth factor 9 (FGF9) and WNT signaling, are more pronounced in large wounds, creating a regenerative niche^152^. In contrast, small wounds fail to recruit sufficient immune mediators and instead exhibit elevated dermal Wnt/β-catenin activity, favoring fibrosis over regeneration. Key molecules like Shh and FGF9 are absent or insufficient in small wounds, disrupting the feedback loops necessary for HF neogenesis^153^. Moreover, small wounds lack the spatial gradients of Wnt ligands observed in large wounds, which seems critical for coordinating regenerative signaling.

Secondly, the spatial scale of large wounds generates distinct microenvironments with pronounced oscillatory gradients of signaling molecules such as Shh and mechanical tension, which can activate stem cells such as papillary fibroblast and promote differentiation into regenerative structures like HFs^37,45,46,101^. Studies suggest that upper wound fibroblasts, enriched in large wounds and marked by the differentiation regulator CRABP1 expression, share transcriptomic similarities with papillary fibroblasts known to support HF morphogenesis^57,154^. Finally, the larger wound area may recruit a greater number of extrafollicular mesenchymal progenitors, such as those marked by Hic1, which contribute significantly to the regenerative dermis and neogenic HFs^155^. These factors collectively create a permissive environment, setting the stage for phenomena like WIHN.

### Wound-induced hair follicle neogenesis (WIHN)

WIHN is a rare regenerative phenomenon in adult mammals where large full-thickness wounds (> 1 cm² in 3-week-old mice) trigger de novo HF formation. The newly formed HFs demonstrate the ability to transition through all phases of the hair cycle, from the anagen (growth) to the telogen phase (quiescent)^156^.

In the center of large wound in the remodeling phase (from day 21), immune cells such as γδT cells and macrophages are activating Wnt/β-catenin signaling in dermal fibroblasts, driving dermal papilla formation and epithelial-mesenchymal crosstalk^152^. Concurrently, CX3CR1+ macrophages release TNF-α and TGF-β1, activating AKT/β-catenin pathways in Lgr5+ HFSCs to promote proliferation and differentiation^157^. Additionally, TLR3-mediated sensing of endogenous dsRNA amplifies regeneration via IL-6/STAT3 signaling and RA synthesis, which reprograms keratinocytes and fibroblasts toward embryonic-like states^158^. Neonatal-like papillary fibroblasts expressing Lef1 drive WIHN regenerating functional HF through DP cell differentiation and dermal condensate induction^159^.

The Wnt signaling pathway plays a crucial role in WIHN: while Shh activation is essential for creating an inductive dermal niche, Wnt signaling is equally important for HF regeneration. It has been shown that long-term Wnt activation in dermal fibroblasts is associated with fibrosis, but when combined with Shh and Blimp1 signaling, it promotes the formation of the dermal condensate and later on the DP, which are required for HF neogenesis^45,160^. In small wounds, where Shh activity is present but insufficient for WHN, Wnt activation redirects fibroblasts toward a fibrotic fate, preventing HF formation^39,77^. A schematic representation of the different players in mammalian wound healing from a spatiotemporal perspective can be found in Fig. 1.

## Spatiotemporal regulation of regeneration

### Hair follicles as pro-regenerative hubs

The regenerative capacity of HFs extends beyond their role in hair growth, positioning them as dynamic orchestrators of scarless wound healing. Recent advances illuminate how molecular crosstalk and stem cell plasticity within HFs enable tissue remodeling^161–164^, while clinical and RNA-seq studies demonstrate their potential to reverse fibrosis in human scars^165,166^. The HF bulge, a reservoir of the HFSC harbors distinct cell subpopulations with unique regenerative capabilities^30,167–169^. For example, Shh secreted by peripheral HF neurons selectively signals to Gli1-expressing HFSC cells to maintain their multipotency. Notably, in healing wounds, these Gli1 perineural bulge cells exhibit lineage plasticity, transitioning into epidermal stem cells to replenish damaged tissue, promote re-epithelialization with scarless healing and facilitate the formation of the epithelium for the neogenic HFs in WIHN^78^. Thus, the spatiotemporally restricted activation of Shh in the proliferative and remodeling phases within the Gli1^+^ progenitor niche of the HF bulge can initiate a regenerative program.

HFs have also been investigated for their role in triggering tissue regeneration. For example, a small clinical study involving the transplantation of HFs into mature scars from human subjects demonstrated that HF transplantation induced epidermal thickening, restored undulating dermal–epidermal junctions, and enhanced vascularization. Notably, the collagen architecture shifted from dense, aligned fibers to a less compact, embryonic-like pattern with reduced collagen I content and suppressed pro-fibrotic factor TGF-β1 and cytokines (IL-13, IL-6). These studies indicate hair follicles themselves are regenerative hubs, orchestrating scarless wound healing through molecular crosstalk, stem cell plasticity, and dynamic remodeling^165^.

### Immune cells and fibroblast regulation drive the continuum between scarless regeneration and repair

The immune system intricately regulates fibroblast and myofibroblast activation during tissue repair and fibrosis, shaping the wound healing trajectory through dynamic cellular interactions. Immune cells, notably macrophages, orchestrate fibroblast behavior via cytokine signaling, direct cell–cell contacts, and microenvironmental cues, driving the fibroblast-to-myofibroblast transition essential for wound closure and ECM remodeling^83^. Macrophages polarize into pro-inflammatory M1 or anti-inflammatory M2 phenotypes. M2 macrophages secrete growth factors such as IGF-1 and fibroblast growth factor 2 (FGF-2), supporting tissue remodeling and angiogenesis, which are essential for scarless regeneration, as observed in fetal wounds^170,171^. For instance, early embryonic mouse wounds (day 15) heal without scars due to minimal immune cell recruitment and reduced TGF-β1, a profibrotic mediator, highlighting the regenerative advantage of low inflammation. T cells further modulate outcomes: regulatory T cells (Tregs) secrete IL-10 to suppress inflammation, aiding scarless healing, while γδT cells enhance keratinocyte proliferation via FGF-7 and IGF-1, accelerating repair without fibrosis^172^. In the early inflammatory phase, pro-inflammatory macrophages secrete CXCL1, a chemokine that recruits neutrophils, amplifying inflammation and indirectly priming fibroblast activation^173–175^. Another myofibroblast subset in skin wounds, including adipocyte-macrophage precursors CD301b^+^, which influences fibrosis and aging^32^. Macrophages also secrete key fibrogenic signals Wnt, TGFβ, PDGF, and resistin-like molecule alpha (RELMα) triggering fibroblast differentiation into contractile myofibroblasts^176–179^. These myofibroblasts upregulate scar effectors like osteopontin, which promotes fibrosis, and lysyl hydroxylase 2, which cross-links collagen, enhancing matrix rigidity and resistance to remodeling^180^. Notably, fascia-resident fibroblasts contribute by dragging pre-existing collagen from deep layers into wounds, a process potentially modulated by macrophage-driven inflammation^136^.The WNT signaling pathway also contributes, with immune cells like macrophages upregulating WNT ligands during repair^181^. WNT activation supports fibroblast proliferation and sustains myofibroblast survival, complementing TGF-β-driven differentiation^182^. Aberrant WNT signaling, however, can exacerbate fibrosis by prolonging myofibroblast persistence via CD26 activation^183^. Thus, the interplay between immune cells, especially macrophages, regulates fibroblast fate and ECM deposition, highlighting the role of the immune system role in wound healing beyond inflammation.

### Mechanical tension and tissue remodeling

Mechanotransduction, a spatiotemporally regulated mechanism that modulated cellular behavior, ECM composition, and signaling pathways, precisely influences tissue repair and remodeling. Its effects on wound healing are significant and recent research is demonstrating its influence on whether healing results in scarless regeneration or fibrotic repair^184–187^. One of the first insights into how mechanical tension affects wound healing comes from the African spiny mouse (*Acomys*), which exhibits enhanced regenerative capacity compared to common mice or humans^184,186^. Research has suggested that in spiny mice, low tension supports scarless healing, enabling the restoration of skin appendages and a flexible, basket-weave ECM pattern. In contrast, high tension in scar-prone species, such as laboratory mice, promotes fibrotic repair, characterized by dense, parallel collagen fibers and myofibroblast activity, yielding fibrosis and scarring^188,189^. Some of the mechanisms involves sustained ERK signaling affecting ECM tension that reduces mechanical stress on fibroblasts, thereby reducing collagen deposition^190,191^. The spiny mouse exhibits remarkable scar-free regeneration, particularly in ear pinna repair, forming a blastema, a proliferative cell mass critical for regenerating skin, hair follicles, and cartilage. Spatial transcriptomics reveal that blastema formation is asymmetric, driven by the proximal wound side, with key genes such as *Lum* and *Twist2* highly expressed from 10 days post-injury. *Lum*, marking blastema and epidermal basal cells, supports ECM remodeling, while *Twist2* aids mesenchymal differentiation. Other genes, such as *Col1a2* and *Fn1*, indicate fibroblast activity in the blastema, contributes to cartilage and dermis regeneration. Immune-related genes, including *Cd209a* and *Clec4g*, are enriched in macrophages, suggesting a pro-regenerative immune response. In contrast, laboratory mice lack blastema formation, showing elevated *Col1a1* and dense collagen deposition, leading to scarring. Acomys’ compliant ECM and transient myofibroblast presence prevent fibrosis, unlike Mus, where persistent myofibroblasts drive scar formation^192^.

Recent research on mechanotransduction pathways further clarifies the role of tension. Studies in mice and pigs demonstrate that Yes-associated protein (YAP)-driven mechanotransduction governs scarring versus regeneration. In red Duroc pigs, verteporfin-mediated YAP inhibition prevented scarring, promoted regeneration, and altered fibroblast dynamics via an IL-33/myeloid cell axis, paralleled in human xenograft models^193^. Separately, murine studies identified Engrailed-1-negative fibroblasts (ENFs) as regeneration-competent cells that activate Engrailed-1 (becoming profibrotic EPFs) under mechanical tension. Blocking YAP signaling via verteporfin or genetic knockout redirected ENFs toward regenerative healing, restoring skin appendages and mechanical strength^37,194^. Together, these findings highlight YAP as a conserved regulator of scarring across species, with mechanotransduction inhibitors offering therapeutic potential to shift wound repair from fibrosis to regeneration. Macrophages also amplify fibrosis through mechanical signaling. The FAK–ERK–MCP1 pathway, activated by mechanical forces, increases monocyte chemoattractant protein-1 (MCP1) production, recruiting more inflammatory cells and sustaining fibroblast activation. Inhibiting mechanotransduction, such as through focal adhesion kinase (FAK) knockdown, reduces inflammation and shifts fibroblasts toward a pro-regenerative profile, decreasing scarring^195^.

### Mice to humans wound healing dynamics crosstalk

Despite extensive data from animal models such as mice and pigs, translating findings to human wound healing requires careful consideration due to fundamental physiological differences^196^. In mice, excisional wounds heal predominantly through contraction mediated by the panniculus carnosus, a subcutaneous muscle layer that is absent in humans^197^. In contrast, human wounds rely heavily on re-epithelialization and granulation tissue formation^150^. To better mimic human healing, silicone splints are applied in mouse models to restrict contraction, shifting the healing process toward keratinocyte-driven re-epithelialization. This splinting alters cellular dynamics, reducing keratinocyte proliferation (e.g., lower keratin-6 expression) while increasing macrophage and myofibroblast recruitment, leading to prolonged inflammation and enhanced angiogenesis via elevated vascular endothelial growth factor (VEGF)^198,199^. Molecularly, splinted wounds upregulate pro-inflammatory cytokines (e.g., IL-1β, TNF-α, COX-2, MCP-1) and matrix metalloproteinase-9 (MMP-9), with reduced collagen type III deposition, potentially increasing fibrosis risk^200,201^.

Even though prolonged inflammation and mechanical stress in splinted models introduce translational challenges. These adaptations make splinted mouse models more relevant for studying human-like wound healing mechanisms, though limitations persist^37^.

Another main difference between recent findings in wound healing dynamics in humans and mice is the presence and involvement of fascia in human wound healing. Although humans do not have a clearly visible fascial layer as in mice, recent studies indicate that humans possess similar populations of fascia fibroblasts. These fibroblasts contribute to pathological conditions characterized by myofibroblast activity, such as keloid lesions, hypertrophic scars, and scleroderma, and can also adopt pro-inflammatory phenotypes in autoimmune conditions like psoriasis^24,48,136^.

Single-cell RNA-seq analyses reveal that human fascia fibroblasts follow a differentiation trajectory similar to that observed in mice, transitioning from homeostatic populations to pro-inflammatory fibroblasts, partially regulated by RA signaling, and subsequently to contractile myofibroblasts, mediated by HIF1α-driven hypoxia signaling. This trajectory is critical for the progression from the inflammatory phase to tissue contraction during wound healing. Conserved markers, such as PI16 for fascia fibroblasts and POSTN and collagen genes for myofibroblasts, further support the similarity of fibroblast behavior between species^24^.

Thus, even in the absence of a clearly defined fascial layer in humans, fascia fibroblasts can adopt phenotypes and differentiation trajectories comparable to those observed in mice. These findings suggest a conserved role of fascia fibroblasts in wound healing across species. However, further research is required to fully define these similarities and their implications for human skin pathologies.

### Spatiotemporal clock regulating wound healing processes

Thus far, we have provided evidence illustrating that wound healing is orchestrated by a finely tuned spatiotemporal process that coordinates signaling pathways across the phases of inflammation, proliferation, and remodeling. After introducing the concept of spatiotemporal clocks earlier, we now present two examples, one in mice and another in humans, to demonstrate how this framework explains pathway dynamics.

### Spatiotemporal clocks regulating fibroblast differentiation and ECM dynamics in wound healing

**Fig. 2:**
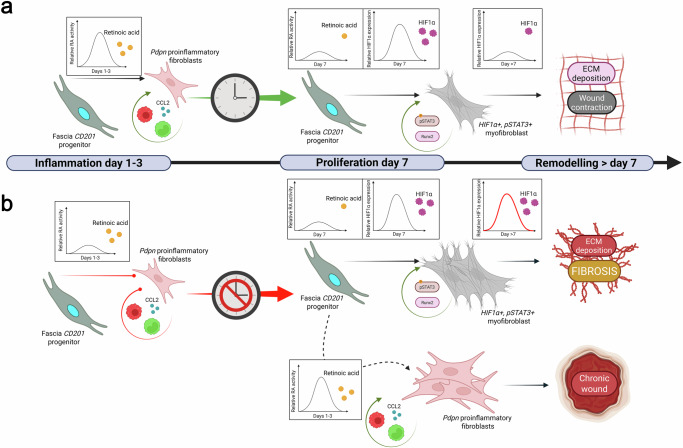
Spatiotemporal clock regulating fibroblast differentiation, scarring and chronic wounds. Retinoic acid and hypoxia-inducible factor 1-alpha (HIF1α) in controlling fibroblast differentiation across wound healing phases. **a** During the inflammatory phase (days 1–3), RA peaks, driving CD201^+^ fascia progenitors to differentiate into pro-inflammatory fibroblasts (Pdpn + ), which upregulate CCL2 to recruit monocytes and macrophages, amplifying inflammation. In the proliferative phase (day 7), RA levels decrease, while HIF1α peaks, promoting the differentiation of CD201^+^ progenitors into pSTAT3^+^ and Runx2^+^ myofibroblasts in hypoxic wound regions, facilitating extracellular matrix deposition and wound contraction. In the remodeling phase (> day 7), both RA and HIF1α decline, supporting ECM remodeling and scar formation. **b** Dysregulation of this clock, such as lack of active RA in the inflammatory phase or prolonged RA or HIF1α activity in the proliferation phase, leads to excessive ECM deposition, resulting in fibrosis or chronic wounds. Graphs above each phase depict the relative expression of RA and HIF1α over time, highlighting their oscillatory dynamics.

Recent research has provided insights into fibroblast fate differentiation and ECM dynamics in wound healing, offering an exquisite model for applying our spatiotemporal clock framework. Retinoic acid (RA) and hypoxia-inducible factor 1-alpha (HIF1α) act as critical oscillators, regulating the differentiation of CD201^+^ fascia fibroblast progenitors into pro-inflammatory fibroblasts and myofibroblasts, respectively^24^. These pathways operate within a spatiotemporal clock framework, synchronizing molecular events with distinct wound healing phases (inflammatory, proliferative, and remodeling) and specific cellular niches to orchestrate orderly phase transitions and determine tissue outcomes and ECM deposition (Fig. 2). In the inflammatory phase (0–3 days), RA signaling peaks, driving CD201^+^ fascia progenitors to differentiate into pro-inflammatory fibroblasts, upregulating chemokines like CCL2 to recruit monocytes and macrophages (Fig. 2a). As the wound progresses into the proliferative phase (3–7 days post-injury), RA signaling oscillates to lower levels, facilitating inflammation resolution, decrease the number of immune cells and gating hypoxia signaling activation through HIF1α. HIF1α peaks during this phase, triggering the differentiation of CD201^+^ progenitors into proto-myofibroblasts and subsequently myofibroblasts. Upon differentiation, myofibroblasts secrete large amounts of collagen and fibronectin while generating contractile forces that reorganize the ECM microenvironment. This dual activity not only restores tissue integrity but also alters the mechanical stiffness and architecture of the wound bed, guiding cell migration and angiogenesis. HIF1α activity is spatially localized to hypoxic regions of the wound, ensuring that myofibroblast differentiation occurs in areas requiring tissue repair^202^. Using HIF1α inhibition and genetic deletion at 7 days post-injury confirms that HIF1α is essential for this contractile phenotype, with dysregulation impairing ECM deposition and delaying wound closure. In the remodeling phase (> 7 days post-injury), HIF1α signaling gradually declines as hypoxia resolves, while RA remains at low levels. This coordinated oscillation of RA and HIF1α levels allows myofibroblasts to sustain ECM remodeling and scar formation, stabilizing the healed tissue. However, prolonged or dysregulated HIF1α signaling in the dermal niche can drive excessive ECM deposition, leading to fibrosis, while insufficient RA signaling in the inflammatory phase may hinder immune responses, resulting in chronic wounds.

RA and HIF1α form a spatiotemporal clock that regulates fibroblast differentiation in the fascia, with RA driving pro-inflammatory fibroblast formation during the inflammatory phase (0–3 days) and HIF1α promoting myofibroblast differentiation and ECM deposition in hypoxic niches during the proliferative phase (3–7 days). This oscillatory behavior characterized by phased, spatially dependent activity ensures that fibroblast differentiation occurs in the appropriate phase and niche, while the ECM attains the correct stiffness.

For example, RA’s peak in the inflammatory phase establishes a pro-inflammatory zone at the wound edge to recruit immune cells, while HIF1α’s peak in the proliferative phase defines a repair zone in the wound bed (Fig. 2a). In pathological scarring, such as hypertrophic scars or keloids, disruptions in the spatiotemporal clock drive excessive fibrosis. For instance, prolonged HIF1α signaling may sustain myofibroblast activation beyond the proliferative phase, promoting excessive ECM deposition, which increases scar. This leads to persistent myofibroblast activation and tissue contraction, contributing to scar formation stiffness (Fig. 2b). The precise coordination of HIF1α, functioning as a molecular oscillator, is critical for balancing proliferation to prevent pathological scarring. In chronic wounds, disruptions in the spatiotemporal clock impair wound healing, leading to delayed closure and ulceration. For instance, deficient RA signaling may prevent pro-inflammatory fibroblast activation, resulting in insufficient inflammatory signaling and delayed wound closure. Conversely, excessive RA signaling during the proliferative phase may sustain inflammation, preventing the recruitment of myofibroblast activity. This dysregulation can lead to lack of tissue contraction, contributing to pathological outcomes such as ulcers. Thus, the precise coordination of RA, functioning as a molecular oscillator, is essential for balancing the inflammatory landscape and promoting progression toward the proliferative phase to promote wound closure. This spatiotemporal clock framework provides a robust model for understanding how RA and HIF1α orchestrate fibroblast differentiation and drive differences between wound healing phases and spatial niches.

### Spatiotemporal clock regulating wound inflammation and epithelization in chronic wounds

**Fig. 3:**
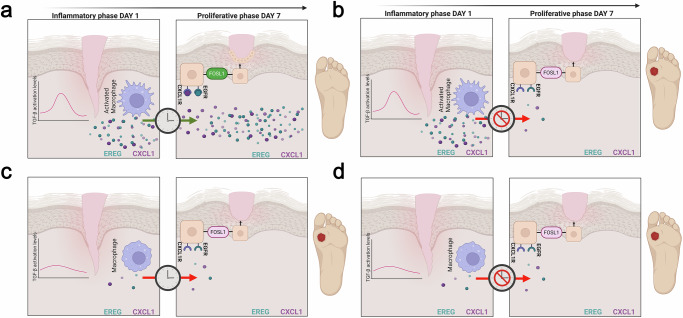
Spatiotemporal clock regulation of CXCL1 and EREG oscillators in wound healing. **a** During the inflammatory phase (day 1), TGF-β signaling activates pro-inflammatory macrophages at the wound edge, releasing CXCL1 and EREG to stimulate local macrophages (purple). When spatial (wound edge) and temporal (day 1 to day 7) coordination of signaling occurs, CXCL1 and EREG bind to their respective receptors, CXCR1 and EGFR, on keratinocytes at the wound margin. This upregulates FOSL1, promoting keratinocyte migration, re-epithelialization, and wound closure in healed wounds. **b** Dysregulated temporal activation of TGF-β signaling prevents sustained CXCL1 and EREG production from day 1 to day 7, impairing their ability to activate keratinocyte receptors (CXCR1 and EGFR) on day 7 due to inadequate threshold levels. This results in failed re-epithelialization, no wound closure, and the development of diabetic ulcers. **c** Lack of spatial activation of TGF-β signaling at the wound edge fails to activate macrophages, leading to insufficient release of CXCL1 and EREG. Regardless of the temporal phase, this prevents signaling to keratinocytes, causing no re-epithelialization and resulting in foot ulcers. **d** Combined lack of spatial and temporal TGF-β signaling disrupts macrophage activation, CXCL1 and EREG release, and subsequent keratinocyte signaling, leading to failed re-epithelialization and foot ulcer formation.

Another example of a spatiotemporal clock can be illustrated through crosstalk between wound inflammation, epithelialization and chronic wounds from a clinical perspective. In humans, during the inflammatory phase, approximately day 1 post-injury, TGF-β signaling activates pro-inflammatory macrophages clustered at the wound edge (Fig. 3). These macrophages release key signaling molecules, such as CXCL1 and EREG, to activate the local microenvironment^53^. By day 7, in the proliferative phase, this spatially localized signaling engages keratinocytes at the wound margin through CXCL1 and EGF receptors, triggering the upregulation of FOSL1 — a transcription factor that drives keratinocyte migration to facilitate wound closure. Concurrently, keratinocytes positioned distally from the wound edge undergo proliferation, establishing distinct zones of cellular activity. The precise coordination of temporal and spatial elements is fundamental here. The timely release of macrophage-derived signals must align with their proximity to the wound edge to effectively activate the appropriate cellular niche, ensuring that keratinocyte migration precedes proliferation triggering successful healing (Fig. 3a). In chronic wounds, such as diabetic foot ulcers, this spatiotemporal coordination is disrupted. Dysregulation in the TGF-β macrophage signaling pathway, spanning both the temporal axis (from day 1 to day 7) and the spatial axis (wound edge), leads to reduced FOSL1 expression in keratinocytes, impairing their migratory capacity and stalling the healing process, ultimately contributing to ulcer formation (Fig. 3b, c). Thus, the interplay of macrophage-derived CXCL1 and EREG with FOSL1 in keratinocytes establishes a spatiotemporal clock that operates during the inflammatory and proliferative phases of wound healing. Here, CXCL1 and EREG act as oscillators within the spatiotemporal clock. Unlike traditional oscillators with rapid, cyclical fluctuations, these molecules exhibit functional oscillation — phased, spatially dependent activity that defines zones: migration near the wound margin and proliferation in distal, healthy areas. This precise coordination drives region-specific responses, underscoring their role in effective wound resolution. Whether a wound heals or becomes chronic hinges on this clock’s proper function. In chronic wound management, for example, therapeutic strategies could be optimized through phase-specific modulation and spatially controlled delivery systems. By precisely coordinating signaling pathways and cellular interactions, such approaches could enhance tissue repair, reduce complications in chronic wounds, and enable phase-specific therapies using advanced delivery mechanisms.

## Conclusions and perspectives

Mammalian wound healing emerges as a dynamic spatiotemporal process, integrating temporal phases with spatially defined tissue domains. Within this framework, we proposed the concept of spatiotemporal clocks — oscillatory regulators that coordinate molecular pathways across defined contexts. These clocks determine whether repair progresses toward regeneration, fibrosis, or chronic non-healing states. By highlighting examples such as RA–HIF1α oscillations in fibroblast differentiation and macrophage-keratinocyte signaling in chronic wounds, we demonstrate how precise temporal and spatial regulation governs phase transitions and regenerative niches.

This perspective reframes wound healing not as a linear cascade but as a programmable system in which timing, spatial alignment, and mechanical forces converge to dictate healing outcomes. Emerging methodologies are now enabling the resolution of these dynamics at unprecedented scales. Future integration with computational modeling could allow predictive mapping of healing trajectories and the rational design of phase-specific interventions, from temporally targeted Wnt agonists to spatially confined modulation of HIF1α or YAP signaling.

Translationally, this framework points toward precision regenerative medicine. Interventions tailored to spatiotemporal dynamics, such as bioengineered scaffolds delivering phase-specific cues or pharmacological modulators of mechanical stress, which could reduce scarring, accelerate chronic wound closure, and restore tissue function. By defining wound repair through the lens of spatiotemporal clocks, we aim to provide a conceptual blueprint for future research and therapeutic innovation. This systems-level perspective has the potential to reshape strategies for trauma, surgical recovery, and chronic wound management, bringing regenerative solutions closer to clinical reality.
